# A fair dividend approach for aggregating wearable sensor data to improve electronic health records

**DOI:** 10.1371/journal.pone.0327942

**Published:** 2025-07-11

**Authors:** Turki M. Alanazi, Noha Alduaiji, Chahira Lhioui, Rim Hamdaoui, Somia Asklany, Monia Hamdi, Ali Elrashidi, Ghulam Abbas

**Affiliations:** 1 Department of Electrical Engineering, College of Engineering, University of Hafr Al Batin, Hafr Al Batin, Saudi Arabia; 2 Department of Computer Science, College of Computer and Information Sciences, Majmaah University, Majmaah, Saudi Arabia; 3 Department of Computer Science and Artificial lntelligence, College of Computing and Information Technology, University of Bisha, Bisha, Saudi Arabia; 4 Department of Computer Science, College of Science and Human Studies-Dawadmi, Shaqra University, Shaqra, Riyadh, Saudi Arabia; 5 Department of Computers and Information Technologies, College of Sciences and Arts Turaif, Northern Border University, Arar, Saudi Arabia; 6 Department of Information Technology, College of Computer and Information Sciences, Princess Nourah bint Abdulrahman University, Riyadh, Saudi Arabia; 7 Electrical Engineering Department, University of business and technology, Jeddah, Saudia Arabia; 8 School of Electrical Engineering, Southeast University, Nanjing, China; Maulana Abul Kalam Azad University of Technology West Bengal, INDIA

## Abstract

Wearable sensor (WS) technology in healthcare is essential because it makes medical diagnosis easier by continuously monitoring important changes in an individual’s body. This technology is used to detect aberrant occurrences and predict medical dangers. A central connecting unit is used to stream and send accurate observations to improve the quality of medical diagnosis. In this paper, we present a Fair Dividend Interrupt Method (FDIM), a new way to arrange and improve the efficiency of combining WS inputs. This approach employs federated learning to prioritize interruptions based on their importance and WS criteria. This leads to well-structured streaming periods across numerous connecting devices, guaranteeing continuous sequences. The sequence determination uses balanced linear scheduling, optimizing the structure of sensing operations and increasing WS input availability when interruptions from multiple sensors, thereby boosting operating efficiency. The proposed approach outperforms baseline methods in access time, computational complexity, data utilization, processing time, aggregation ratio, and error rate by 10.18%, 5.19%, 10.57%, 8.48%, and 10.42%, respectively. Due to these developments, FDIM is now a highly efficient, scalable solution for wearable healthcare systems that allows accurate medical decision-making.

## 1. Introduction

### 1.1. Background

Wearable sensors are one of the emerging technologies in today’s world of automation. These devices are widely used for healthcare monitoring, patient tracking, sports performance analysis and more. They play a vital role patient monitoring systems by providing real-time data that helps identify and assess medical conditions accurately [1]. To enhance the effectiveness of these systems, an improved Bayesian convolution network (IBCN) algorithm is used in wearable sensor devices to enhance the monitoring system and provide better patient service. IBCN helps to analyze the emotions captured by the device and provides an accurate dataset for further diagnosis [2,3]. In particular, the convolution layer is used in wearable sensors for the feature extraction process. Feature extraction is done based on certain features such as the vital signs, heartbeat rate, and audio signs. The convolution layer analyzes the features and separates the features used for the diagnosis process [4].

Wearable Sensor Systems (WSS) contribute significantly to reducing medical costs, by offering timely and efficient patient care. These systems monitor patient activity and conditions, generating sensor signals that support accurate medical diagnoses [2,5]. Moreover, they help in understanding user behavior and facilitate communication between patients and healthcare providers. However, providing accurate details is challenging for every wearable sensor device. To address this, machine learning algorithms are increasly integrated into wearable sensors to optimize performance and accuracy [6]. Devices such as wristwatches, smart applications, smart devices, glasses, and wearable clothes are mostly used in in both healthcare and security contexts [7]. Among the techniques used, sequential minimal optimization method plays a key role in interpreting user activity and improving classification accuracy [8]. Sensor nodes in these systems collect data from devices or network, which is then generated as a signal for further use. Additionally, optimization techniques like the Markov model is used as an optimization process to increase the efficiency and performance of the system by ensuring accuracy and scalability. Feature extraction remains a critical component, significantly improves performance by calculating the actual features based on the stored values from the database [9,10].

Wearable sensors arealso used to improve daily life due to affordability and usability. hey facilitate user-device interaction, including location tracking through sensor nodes [11]. Multi-level decision systems (MDS) further support this by comparing real-time data with stored values for informed decisions [12]. One of the major challenges in the wearable sensor is the energy consumption rate. Charge space accumulation (CSA) is used in sensor devices to reduce energy consumption by creating a shielding layer to prevent the power rate while processing [13,14]. CSA understands the users’ needs and avoids unnecessary requests from the users to the organizations. Beyond healthcare, wearable sensors assist in public safety applications such as crime prevention, where sensor data helps track and identify individuals [10,15]. Additionally, these devices enhance well-being by tracking stress, physical activity, sleep, and nutritional habits- underscoring their potential in promoting holistic health [16]. Electronic Health Records (EHRs), digital databases of patient health information, have been developed for effective medical data administration. Promoting smooth information exchange in a digital health setting is important for leveraging the advantages of electronic health records [17]. In today’s setting, consumers anticipate a smooth flow of data, with the incorporation of time domain data in EHRs essential for identifying trends. This time-series data comes from wearable technology that monitors current health trends [18].

However, traditional approaches to managing wearable sensor data often suffer from latency, poor resource utilization, and high error rates. The Flexible Data Integration Model (FDIM) addresses these limitations through interrupt-driven multi-bridging, priority-aware scheduling, and adaptive streaming. FDIM significantly improves data throughput, reduces computational and processing delays, and enhances energy efficiency. Experimental results substantiate the quantitative benefits of FDIM shows a 10.18% decrease in access time, a 5.19% reduction in computational complexity, a 10.57% increase in data utilization, an 8.48% decrease in processing time, a 9.97% enhancement in aggregation ratio, and a 10.42% reduction in error rates across diverse priority probabilities. FDIM significantly improves data throughput, inference delay, and power efficiency compared to current federated learning-based aggregation approaches, establishing it as a better solution for scalable and adaptable medical data processing. These benefits position FDIM as a formidable and efficient framework that integrates accuracy, efficiency, and real-time flexibility in wearable healthcare systems.

### 1.2. Motivation and contribution

However, traditional methods of data integration have substantial limitations, despite the rapid proliferation of wearable sensors in healthcare and public safety, which has transformed real-time monitoring. On the other hand, when it comes to managing time-series data from dispersed sensor nodes, they include excessive energy consumption, delay, inefficient resource utilization, and increasing error rates. On a large scale, existing models frequently struggle to maintain accuracy and efficiency, and they often cannot respond adaptively to sensor priorities. As a result, there is an urgent requirement for a data integration framework that is not only adaptable but also real-time and low-latency. This framework should be able to manage large-scale sensor data streams that are dependent on the passage of time in wearable systems.

In this study, the most important contributions are as follows:

1. The Fair Dividend Interrupt Method (FDIM) is a novel approach that aims to increase the efficiency of combining WS inputs by arranging them more efficiently.
2. To enhance the quality of medical diagnosis, accurate observations are transmitted through a central connecting unit and broadcast to the relevant parties.
3. Federated learning assigns a priority to interruptions according to the seriousness of the interruptions and the WS criteria.
4. A number of parameters, such as access time, complexity, data utilization, processing time, aggregation ratio, and error, are given an evaluation.

The rest of the paper is structured as follows: Section 2 reviews related work, Section 3 details the proposed Fair Dividend Interrupt Method (FDIM), Section 4 presents results and discussion, and Section 5 concludes the study.

## 2. Related work

Wang et al. [19] proposed a cardiovascular healthcare system based on the Internet of Medical Things (IoMT), utilizing a wearable electrocardiogram (ECG) patch to monitor patient activity. This wireless patch enhances system optimization by reducing radio noise during ECG signal transmission and improving prediction accuracy before formal diagnosis. Similarly, Qiu et al. [20] introduced a multi-sensor fusion approach using a Body Sensor Network (BSN) for healthcare monitoring. Their method analyzes medical data through gait features, employing wearable Inertial Measurement Units (IMUs) to accurately extract user-specific characteristics. Gait assessment enhances early diagnosis, contributing to reduced mortality rates. Compared to existing methods, the proposed approach significantly improves the performance of the monitoring system.

Manogaran et al. [21] introduced a cognitive data processing method for the uncertainty analysis process (CDP-UA) to enhance the performance of wearable sensors. CDP-UA consists of two stages: dissemination and aggregation. A classification algorithm is emplyed to manage mapping process between dissemination and aggregation, while a joint learning algorithm helps reduce the latency rate and improves user communication. Experimental results show that the proposed CDP-UA method reduces the time consumption rate and improves the system’s overall performance.

Liu et al. [22] proposed a transmission rate adaption-assisted energy-efficient allocation scheme for wireless body area networks (WBAN). The proposed method aims to improve the Quality of Services (QoS) by categorizing the exact features of the network. A priority-based retransmission strategy (PRS) approach is used in the retransmission process to identify key requirements to improve the QoS. Compared to existing schemes, the proposed approach proves more effective in enhancing overall service quality.

Alqahtani et al. [23] proposed a divergence-dependent transmission scheme for medical applications. Based on the recurrent feedback analysis process, free channels are identified. Sensed signals are transmitted via overlapping channels. The diagonal analysis process is used to identify the noise migration based on the assessed feedback channels. Experimental results show that the proposed method improves overall performance by providing a better communication process than the existing schemes.

Xie et al. [24] proposed a data assessment method for healthcare system based on multi-vital biosignals. Internet of Things (IoT) is widely used in healthcare applications, which helps to provide better services to patients at a needed time. IoT collects user biosignals and stores them in the database for further use. The proposed method reduces latency in accessing healthcare services and shortens patients’ waiting time for diagnosis. It also recommends nearby healthcare centers for timely service delivery. Lalouani et al. [25] introdcuced an energy-efficient data collection method for wearable sensors. This method is used to reduce the latency rate and energy consumption rate of the network. A data quantization approach is used to increase the network’s throughput and service accuracy rate. Additionally, a packet formation algorithm minimizes the energy consumption rate and improves the network bandwidth. The proposed method increases effectiveness and improves prediction accuracy.

Alsiddiky et al. [26] introduced a priority-based data transmission process using selective decision modes for wearable healthcare devices. Healthcare monitoring systems play a vital role in wearable sensor devices. The proposed method improves the data transmission process and avoids data losses via transmitting data. The proposed method uses the queuing approach to analyze the users’ requests and help provide appropriate patient services at the required time.

Rashid et al. [27] proposed a cooperative, reliable, and energy-efficient routing protocol (Co-REERP) for intrabody sensor networks (Intra-WBSN). Biosignals are collected with the help of an electrocardiogram (ECG) and directly transmitted to the body network controller (BNC) for the classification process. Sensor nodes help to improve the healthcare monitoring system. The proposed method increases energy efficiency and improves the system’s performance compared to the existing methods. Kammerdiner et al. [28] introduced a data-driven combinatorial optimization for sensor-based assessment for a 5G network. This method is used in disease detection and improves the accuracy rate in wearable sensor devices. Experimental results show that the proposed method improves overall performance, increases efficiency, and reduces latency in healthcare applications.

Ben-Romdhane et al. [29] proposed a data transfer optimization process using an event-driven approach for healthcare applications. Electrocardiogram (ECG) signals are used in the proposed method for the data transmission process. The proposed method uses a level-crossing analog-to-digital convertor (LC-ADC) to find out the exact signals that ECG generates, which helps to give a better diagnosis process to the users. Compared with other methods, the proposed scheme reduces the network’s cost and time consumption rate. Karmakar et al. [30] proposed a fault detection and recovery framework for remote healthcare monitoring systems using a wireless body area network (WBAN). Patients’ healthcare details are transmitted as a signal to the healthcare centers, which is used in the diagnosis process. This method is also used to identify the fault sensor nodes and helps to reduce the storage space. Experimental results show that the proposed framework increases reliability and accuracy and reduces the time consumption rate while providing user services.

Khan et al. [31] proposed a fragmentation-based media access control (MAC) scheme (FROG-MAC) for wireless sensor networks (WSN). FROG-AMC improves overall latency rate and reduces the traffic rate in the WSN system. Experimental results demonstrate that the proposed FROG-MAC method increases the overall performance and reduces the time consumption rate while processing services to users compared to existing schemes. Faisa et al. [32] introduced TVA (Time-Varying Analytics), a secure and flexible time series evaluation system that utilizes outsourced MPC. TVA safeguards data by spreading trust and extends previous efforts’ capabilities without compromising security or performance. To reduce the strain between both of these, TVA uses innovative procedures, optimizations across several layers, and vectorized basic elements that allow it to handle big datasets with limited usage of resources. Allioui, H., & Mourdi, Y. [33] examined the possibility of IoT for improved financial growth and stability, with a specific emphasis on IoT management of data, connectivity, IoT data analytics, IoT data privacy, as well as other pertinent factors that influence the IoT environment. Their study offers a comprehensive analysis of current IoT research across multiple domains and serves as a valuable guide for researchers and practitioners to identify emerging opportunities and drive future advancements in the field.

Xiaoding Wang et al. [34] suggested Hierarchical Federated Learning for Accurate Anomaly Detection in the Industrial Internet of Things. The goal of federated learning is to construct a global anomaly detection model using the deep reinforcement learning (DRL) method to train individual local models. There is less risk of privacy leakage during federated learning since local data sets are not needed. Detection design and the author’s introduction of privacy leakage degree may also substantially enhance detection accuracy. According to validation trials, the suggested approach positively impacts latency and anomaly detection accuracy. These results bode well for privacy protection across a range of IIoT applications.

Xiaoding Wang et al. [35] proposed the Blockchain-Empowered Internet of Things for Secure Data Aggregation Strategy in Edge Computing. Mobile data collectors (MDCs) and other task receivers may be limited in their ability to search for and accept tasks by adding a security label to the block header that specifies the job’s security level (SL) and the criteria for its completion. New rules for block creation have been created to enhance the system’s performance regarding transaction latency and throughput. In addition, BSDA protects against privacy exposure by breaking out sensitive jobs and task recipients into categories. While data aggregation tasks often have lower SLs, an improved self-adaptive double bootstrapped deep deterministic policy gradient (IDDPG) is a deep reinforcement learning approach that may be used to construct energy-efficient MDC routes.

Wang Xiaoding et al. [36] proposed the Heterogeneous Blockchain and AI-Driven Hierarchical Trust Evaluation (BHTE) for 5G-enabled Intelligent Transportation Systems (ITS). The hierarchical incentive systems ensure that incentives and penalties are rational and fair, and the trust between ITS users and task distributors is evaluated using federated deep learning. ITS users’ and task distributers’ trust is kept on hierarchical and diverse blockchains to verify trust. The comprehensive experimental findings demonstrate that (i) the proposed BHTE framework provides fair and accurate trust evaluations and (ii) the BHTE performs efficiently with low latency and great system throughput.

Mehta and Patel [37] offer CMAF-IIoT, a Chaotic Map-Based Authentication Framework, to solve IIoT security concerns. The main issue is the lack of lightweight, robust, and scalable authentication solutions for resource-constrained IIoT systems, which are routinely attacked by sophisticated assaults. The authors produce random and safe authentication tokens using elliptic curve encryption and a chaotic map function. Formal security analysis and performance evaluation on compute time, energy usage, and communication overhead are included. The method dramatically reduces authentication delay and improves replay and impersonation resistance. The framework’s dependence on pre-distributed keys and lack of dynamic network topology deployment and testing are drawbacks. The report recommends more research to improve flexibility in highly mobile IIoT networks and test scalability in large-scale implementations.

Karia and Rana [38] develop the PUF and Authenticated Encryption-Based Authentication Framework to secure IoT-enabled smart healthcare systems. The research addresses illegal access and data manipulation in sensitive medical situations where typical encryption approaches are too computationally intensive. Physically Unclonable Functions (PUFs) with authenticated encryption guarantee secure and lightweight identity verification and data sharing. Protocol design, simulation-based performance analysis, and AVISPA security validation are their methods. PAAF-SHS is efficient in overhead communication and resistant to man-in-the-middle and eavesdropping. The framework’s reliance on static device settings and limited testing on dynamic healthcare scenarios like mobile health or emergency response systems are drawbacks. Future research should include adaptive security provisioning and machine learning anomaly detection in real-time healthcare monitoring.

Sharma et al. [39] offer a resource-efficient and secure data transfer technique for restricted smart wearable devices. Inefficient use of processing and network resources during safe data exchange can compromise wearable devices’ real-time health monitoring capability. The authors present a lightweight authentication and data sharing protocol using elliptic curve integrated encryption and hash-based message authentication codes. For IoT contexts, they use NS-3 and Contiki OS for mathematical modeling, protocol creation, and simulation. The mechanism improves energy efficiency, communication cost, and delay. The restricted focus on symmetric cryptography and static key distribution may not scale well for large or heterogeneous device networks. The system has not been evaluated in multi-hop or federated learning-based wearable networks, limiting its real-world usefulness in different mobility and data fusion scenarios.

## 3. The proposed fair dividend interrupt method

**Fig 1:**
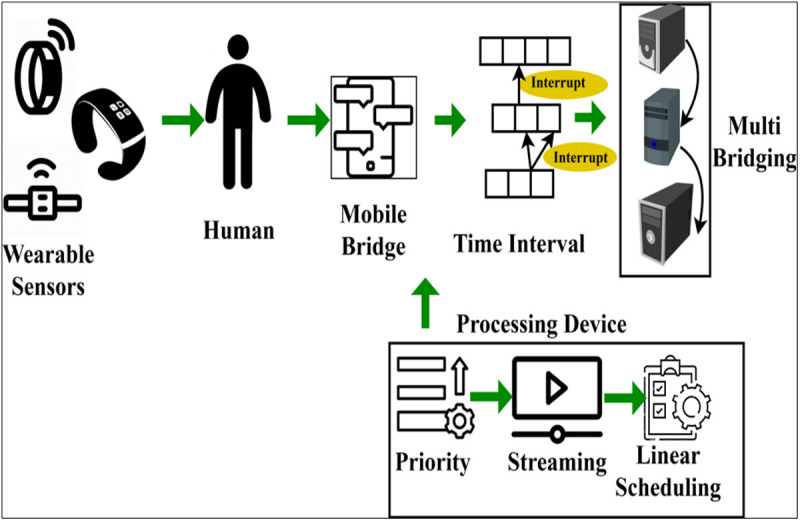
Proposed method.

The proposed smart biomedical technology is designed to play an important role in the healthcare system medical diagnosis functions through smart biomedical instruments. In a smart biomedical scenario, medical diagnostic devices such as Fingertip Pulse Oximeter, EEG, ECG, etc., help monitor or measure various health parameters. These diagnostic monitors are a combination of software and hardware components used to process and organize information from the healthcare industry. The healthcare monitor includes a wearable sensor to collect information such as heartbeat rate, pulse level, brain waves, etc. This data is used to organize data from wearable sensors and manage the operations of the medical diagnosis devices. In the proposed work, the FDIM improves the evaluation of exact information and the accuracy of diagnoses through the aggregation of processing devices. Fig 1 presents an illustration of the proposed method in the healthcare scenario. The FDIM is designed to improve the accumulation of streaming/transmitting exact information from the organization based on wearable sensor data inputs. These inputs are collected from the human body at regular intervals based on key physiological changes. The accumulated data is analyzed using a multi-bridging processing device for precise health record augmentation (Fig 1).

Table 1 describes aberrant event detection, medical risk prediction, and federated learning-based scheduling. To optimise data streaming, error estimates, and diagnosis across dynamic sensor observations, normalised input, deviation functions, and classification labels are crucial. The main goal of this technology is to reduce error factor and interrupt ratio observing from medical diagnosis. The challenging role is the maximization and connection of observing sequences with the existing instances. The observing sequence is organized using multi-bridging devices from the WS specifications. The WS inputs can be of any type based on identifying pulse levels, brain waves, etc. In a WS input instance, the input received $\left({WS}_{i}\right)$ is derived as

**Table 1:** Notation and its description.

Notation	Description
${WS}_{i}$	Input instance from the wearable sensor
$M_{D}$	Set of active wearable sensor data
${M_{D}}_{\max}$	Maximum WS data observed over intervals
${M_{D}}_{\min}$	Minimum WS data observed over intervals
ϑ	Abnormal event detection threshold
$𝒩\left({WS}_{i}\right)$	Normalized WS input instance
α	Detected abnormal event count
β	Predicted medical risk factor based on sensor behavior
$a^{k}$	Streaming interval instance; part of input data sample
$b^{k}$	Label for input $a^{k}$ ; + 1 = streaming condition, −1 = non-streaming
A	Set of all data input samples
$\overset{―}{A}$	Subset of A where $b^{k}$ = + 1 (streaming or interrupt events)
$\overset{―}{B}$	Subset of A where $b^{k}$ = −1 (non-streaming or silent intervals)
g:A→B	Mapping function for classifying samples
γ	Diagnosis accuracy factor
\Updelta	Sigmoid deviation function controlling scheduling behavior
$g_{1}$ , $g_{2}\ldots .g_{n}$	Priority-checked scheduled output values over instances
n	Total number of instances or time intervals


$$\begin{array}{c} {WS}_{i}=M_{D}\frac{\times ({M_{D}}_{\max}-{M_{D}}_{\min})}{M_{D}}+{M_{D}}_{\min}\\ \text{such that}\\ \vartheta =1/{\left(2\pi \right)}^{2}\left[\frac{\left(\frac{{M_{D}}_{\min}}{{M_{D}}_{\max}}-\frac{M_{d}}{M_{D}}\right)}{2\left({WS}_{i}-\tilde{{WS}_{i}}\right)}\right] \end{array}\}$$ (1)

Where, $M_{D}$ is the active wearable sensor input and $M_{d}\in M_{D}$ , ${M_{D}}_{\max}$ and ${M_{D}}_{\min}$ are the minimum and maximum WS data observed in different time intervals. The variable ϑ and $\tilde{{WS}_{i}}$ are helps to represent the detecting of abnormal events and predict medical risks. The detection of abnormal events is computed as the total number of incongruous instances accessed at any different $M_{D}$ observations. There are some conditions of error factors in $M_{D}$ based on abnormal events and medical risks of $M_{D}$ . Therefore, these problems affect the $M_{D}$ at any instance, for which the normalization of vital changes in the human body at regular intervals is estimated as


$$\begin{array}{c} 𝒩\left({WS}_{i}\right)=\frac{\alpha^{2}}{\alpha^{2}{\left(\frac{{M_{D}}_{\min}}{{M_{D}}_{\max}}-\beta \right)}^{2}}\\ and\\ \beta =\frac{1}{M_{D}}\sqrt{\frac{1}{M_{d}-1}\sum_{i=1}^{M_{D}}{\left(\frac{{WS}_{i}-\tilde{{WS}_{i}}}{{WS}_{i}}\right)}^{2}} \end{array}\}$$ (2)

From the above equation (2), the normalization of ${WS}_{i}$ , follows the maximum detection of abnormal events α and the prediction of medical risks β . Here, β is a way to identify the medical risks, and α is the abnormal event detection, for which the accurate computation of ${WS}_{i}$ is observed. Based on ${WS}_{i}$ and $𝒩\left({WS}_{i}\right)$ , the sequence of these error factors is estimated as


$$\exists \left[{WS}_{i},𝒩\left({WS}_{i}\right)\right]=\sqrt{{\left[\frac{𝒩\left({WS}_{i}\right)}{{WS}_{i}}\right]}_{1}^{2}+{\left[\frac{𝒩\left({WS}_{i}\right)}{{WS}_{i}}\right]}_{2}^{2}+\ldots +{\left[\left(1-\frac{𝒩\left(\tilde{{WS}_{i}}\right)}{{WS}_{i}}\right)\alpha \right]}_{M_{d}}^{2},M_{d}\in M_{D}}$$ (3)

Equation (3) denotes the error factor ∃ in this healthcare industry as a consequence of observation until $M_{d}$ is active in pursuing the healthcare process. The pursued information maintaining of the control depends on the time interval until the medical diagnosis requires a service such as streaming/transmitting. The above instance of aggregation efficiency is analyzed using federated learning. In a smart healthcare medical diagnosis scenario, the sensed information must be transmitted into precise observations.

**Fig 2:**
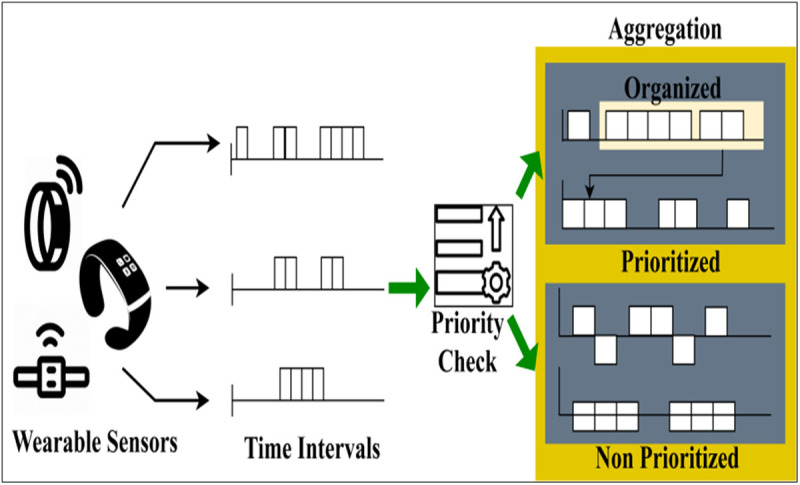
Data aggregation process.

A smart biomedical environment consists of a centralized bridging unit responsible for streaming/transmitting precise observations of smart biomedical instruments and streaming intervals. The operation of the instruments is managed using key changes in the human body and WS inputs. The FDIM method functions between abnormal events and predicting medical risks. The working of the device is maintained by the bridging unit, where smart evaluation and intelligent prediction are made. Predicting precise observations improves the accuracy and efficiency of WS input in diagnosis. Fig 2 illustrates the data aggregation process from the WS.

Feature alignment uses statistical matching and dynamic time warping (DTW) to synchronize diverse sensor data streams. To make sure that different sensor resolutions and sampling rates are consistent, normalization uses min-max scaling and z-score standardization. Using a federated learning architecture, adaptive weighting methods, and differential privacy-preserving aggregation, FDIM resolves feature distribution disparities across devices. To address problems with non-IID data, model changes are synchronized worldwide using federated averaging (FedAvg) and momentum-based optimization while being trained locally on device-specific distributions.

The inputs shared in different time intervals are first classified for their priority. Based on the priority, the organization is performed. In this organization process, $M_{d}$ and v is validated to improve the classification ratio. This is required to leverage the aggregation ratio and mitigate deviation and errors (Refer to Fig 2).

The deployed wearable sensors sense healthcare industry information from smart biomedical diagnoses. The bridge unit is a way to transmit information from wearable sensors to processing devices. Besides, the spread control is to be instantly to meet the healthcare/medical diagnosis requirements. Therefore, using federated learning, the aggregation efficiency of WS inputs helps with ∃ estimation. We consider transmission or streaming of information issues, let the characteristic represented by $a^{k}$ until for the $k^{th}$ characteristics, the corresponding streaming of labels $b^{k}$ from the space label from the sensing organization. Let the corresponding characteristics of the efficiency of WS inputs be denoted by $\overset{―}{A}$ and $\overset{―}{B}$ , respectively.


$$\begin{array}{c} \overset{―}{A}=\left\{a^{k}\in A\right\}:\left\{b^{k}=+1\right\}\\ and\\ \overset{―}{B}=\left\{a^{k}\in A\right\}:\left\{b^{k}=-1\right\} \end{array}\}$$ (4)

For any ${a^{k}}^{+}\in A$ and ${a^{k}}^{-}\in A$ , the objective of this transmission is to build an operation g:A→B such that


$$\begin{array}{c} g\left({a^{k}}^{+}\right)=+1\\ and\\ g\left({a^{k}}^{-}\right)=-1 \end{array}\}$$ (5)

For our given condition of predicting medical risks and abnormal event detection, we represent the condition of dividing interrupts and streaming intervals as labels $b^{k}=+1$ , and the condition of non-dividing interrupts and non-streaming information as $b^{k}=-1$ . Based on this condition, the complete data can be shared for streaming of ${WS}_{i}$ at different streaming interval instances of $a^{k}$ and $b^{k}$ are the root of the WS input for the federated learning process. For an ∃ estimation, the consequence of precise observation is computed as


$$\begin{array}{c} \begin{array}{c} a^{k}={WS}_{i}\\ b^{k}=0 \end{array}\},\text{as the initial data is observed}\\ \begin{array}{c} a^{k}=𝒩({WS}_{i})\\ b^{k}=\frac{\beta }{\alpha } \end{array}\},\text{for the consequence of data}\\ \begin{array}{c} \text{and }\\ a^{k}+b^{k}={WS}_{i},\text{is the initial data sample}\\ \text{and }𝒩\left({WS}_{i}\right)+\frac{\beta }{\alpha },\text{is the input for consecutive information} \end{array} \end{array}\}$$ (6)

Hence, in federated learning, the sequence of $a^{k}+b^{k}=𝒩\left({WS}_{i}\right)+\frac{\beta }{\alpha }$ data can be observed and organized in sensor instances. This learning consists of two methods: priority checking and linear scheduling, followed by the result. The multi-bridging ∃ operations and their diagnosis accuracy treated by federated are defined per the equation (7).


$$\begin{array}{c} M^{bg}\left\{\exists \left[{WS}_{i},𝒩\left({WS}_{i}\right)\right]\right\}=-a^{k}g-b^{k}{WS}_{i}-g{WS}_{i}\mathrm{\backslash Updelta}\\ \text{such that}\\ \begin{array}{c} a^{k}\left(g|{WS}_{i}\right)=\gamma ({WS}_{i}+\mathrm{\backslash Updelta}g)\\ b^{k}\left({WS}_{i}|\alpha \right)=\gamma (a^{k}-\mathrm{\backslash Updelta}g) \end{array} \end{array}\}$$ (7)

**Fig 3:**
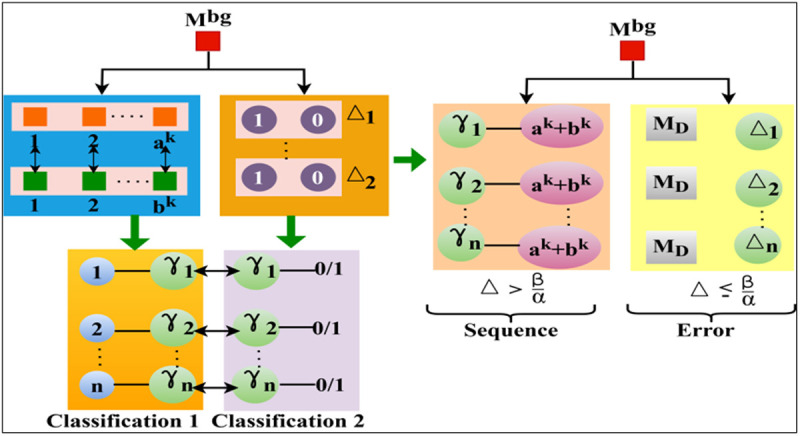
Classification process.

In the above equation (7), $a^{k}\left(g|{WS}_{i}\right)$ and $b^{k}\left({WS}_{i}|\alpha \right)$ are the observed features that are used for satisfying the conditions of the $\exists \left[{WS}_{i},\;N\left({WS}_{i}\right)\right]$ . As per the streaming of $a^{k}\left(g|{WS}_{i}\right)$ and $b^{k}\left({WS}_{i}|\alpha \right)$ , the linear scheduling of α and $𝒩\left({WS}_{i}\right)$ jointly gives the output of $\exists \left[{WS}_{i},\;N\left({WS}_{i}\right)\right]$ at its nearest possible conjunction. During the time interval, interrupts can occur, and the sequence of interrupts can be divided using FDIM. The FDIM is a method of dividing interrupts based on priority and WS specifications using federated learning. Each of the possible transmission information and its features can be analyzed using the federated learning process executed for $n*\alpha_{i}$ observation. The learning process for different classifications is presented in Fig 3. In the first classification, the condition $\mathrm{\backslash Updelta}>\frac{\beta }{\alpha }$ and the second classification relies on $\mathrm{\backslash Updelta}\leq \frac{\beta }{\alpha }$ is administered. In the further processes, the \Updelta from 1 to n instances are assessed for γ . Contrarily, in the derived sequence post-classification, the deviations are performed as sequence and error, as illustrated in Fig 3.

Adaptive client weighting and customized model aggregation approaches are used to mitigate the effect of non-IID data on federated learning convergence. Grouping clients with comparable data distributions improves local model generalization in FDIM’s clustered federated learning technique before global aggregation. Stable updates across heterogeneous wearable sensor data are ensured using FedProx-based regularization to prevent local model drift. Dynamic batch normalization is also used to match feature distributions across devices further and reduce statistical inconsistencies. The resilience of FDIM for real-world deployment is confirmed by an empirical evaluation of convergence stability, which measures gradient divergence and model correctness over different degrees of non-IID situations. Therefore, the deviations in different time instances are identified based on changes in the above classifications. Therefore, the error from $M^{bg}$ is isolated to prevent further deviations. In contrast, the succeeding sequences are prevented from entering the above condition, and hence, the validations are performed without $\exists \left[{WS}_{i},\;N\left({WS}_{i}\right)\right]$ . Now, as represented in the above equations, the first instance and then further consecutive instance, the multi-bridging properties are as defined in equation (6), and hence, (α=β)=0 is the result of the next properties and, therefore, the wearable sensor value of ${WS}_{i}$ is retained without precise information.

Using equation (8), the \Updelta sigmoid function and its associated estimations of $a^{k}$ , $b^{k}$ merging to g is given as


$$\begin{array}{c} \mathrm{\backslash Updelta}=1-\frac{R_{b^{k}}}{R_{a^{k}}}\\ \text{such that}\\ \begin{array}{c} a^{k}\left(g|{WS}_{i}\right)\text{switch to }{WS}_{i},\text{ if }\mathrm{\backslash Updelta}>\frac{\beta }{\alpha }\\ \begin{array}{c} \text{else}\\ a^{k}\left(g|{WS}_{i}\right)\text{switch to }\beta or\alpha ,\text{ if }\mathrm{\backslash Updelta}\leq \frac{\beta }{\alpha } \end{array} \end{array} \end{array}\}$$ (8)

Where the variables $R_{b^{k}}$ and $R_{a^{k}}$ denotes the observations of $a^{k}$ and $b^{k}$ to the possible solutions. It is to be pointed out that not complete data can be observed with both $a^{k}$ and $b^{k}$ . Now, the priority checking output for the $\mathrm{\backslash Updelta}>\frac{\beta }{\alpha }$ and $\mathrm{\backslash Updelta}\leq \frac{\beta }{\alpha }$ conditions are computed as in equation (9)

Condition 1: $\mathrm{\backslash Updelta}>\frac{\beta }{\alpha }$

Priority Checking:


$$\begin{array}{c} g_{1}=𝒩{\left({WS}_{i}\right)}^{1}\\ g_{2}=𝒩{\left({WS}_{i}\right)}^{2}-{\left(\frac{\beta }{\alpha }\right)}^{1}-{\left(\frac{\exists }{M_{D}}\right)}^{1}\\ \begin{array}{c} g_{3}=𝒩{\left({WS}_{i}\right)}^{3}-{\left(\frac{\beta }{\alpha }\right)}^{2}-{\left(\frac{\exists }{M_{D}}\right)}^{2}\\ g_{n}=𝒩{\left({WS}_{i}\right)}^{n}-{\left(\frac{\beta }{\alpha }\right)}^{n-1}-{\left(\frac{\exists }{M_{D}}\right)}^{n-1},n\in \text{instances} \end{array} \end{array}\}$$ (9)

Condition 2: $\mathrm{\backslash Updelta}\leq \frac{\beta }{\alpha }$

Priority Checking:


$$\begin{array}{c} g_{1}={\left({WS}_{i}\right)}^{1}-b^{k}{\left({WS}_{i}|\alpha \right)}^{1}\\ g_{2}={\left({WS}_{i}\right)}^{2}-b^{k}{\left({WS}_{i}|\alpha \right)}^{2}-{\left(\frac{\mathrm{\backslash Updelta}*\exists }{M_{D}}\right)}^{1}\\ \begin{array}{c} g_{3}={\left({WS}_{i}\right)}^{3}-b^{k}{\left({WS}_{i}|\alpha \right)}^{3}-{\left(\frac{\mathrm{\backslash Updelta}*\exists }{M_{D}}\right)}^{2}\\ g_{n}={\left({WS}_{i}\right)}^{n}-b^{k}{\left({WS}_{i}|\alpha \right)}^{n}-{\left(\frac{\mathrm{\backslash Updelta}*\exists }{M_{D}}\right)}^{n},n\in \text{instances} \end{array} \end{array}\}$$ (10)

The derived priority checking result follows for n instances where the transmission and ${WS}_{i}$ are the incrementing factors for deriving the output of the linear scheduling process. The data can be streamed priority-wise and processed using the linear scheduling function over the WS specifications. The data can be checked priority-wise while others are held and then checked for priority. This looping process is continuously performed at different time intervals. This requires linear scheduling of g and \Updelta estimating the further sequence of data.


$$\begin{array}{c} \begin{array}{c} M^{bg}\left\{\exists \left[{WS}_{i},𝒩\left({WS}_{i}\right)\right]\right\}=\left({WS}_{i}+\mathrm{\backslash Updelta}g\right)g-\left(\beta -\mathrm{\backslash Updelta}g\right){WS}_{i}-g{WS}_{i}\mathrm{\backslash Updelta}\\ =-{WS}_{i}g-\mathrm{\backslash Updelta}g^{2}-\beta {WS}_{i}\\ \left\{ \begin{array}{c} 2{M_{D}}_{\min}𝒩\left({WS}_{i}\right)-\mathrm{\backslash Updelta},\text{ if }M_{d}=M_{D}\text{ and }{M_{D}}_{\max}=0\\ -2{M_{D}}_{\max}𝒩\left({WS}_{i}\right)-\mathrm{\backslash Updelta}=\frac{\rho_{\gamma }}{\rho_{\beta }}-2{M_{d}}_{\max}𝒩\left({WS}_{i}\right)-1=-2{M_{D}}_{\max}𝒩\left({WS}_{i}\right),\text{ if}\frac{\rho_{r}}{\rho_{\beta }}=1, \end{array}\right. \end{array}\\ {M_{d}}_{\min}=0,\text{ and }M_{d}=M_{D} \end{array}\}$$ (11)

The above equation (11) denotes the linearity scheduling in ${WS}_{i}$ observed as ${WS}_{i}b^{k}-{{WS}_{i}}^{2}$ is an output for following abnormal events. Now, the following next instances for $M^{bg}\left\{\exists \left[{WS}_{i},\;N\left({WS}_{i}\right)\right]\right\}$ is designed for the priority and WS specifications output for the condition $\mathrm{\backslash Updelta}\leq \frac{\beta }{\alpha }$ as estimated as


$$\begin{array}{c} M^{bg}\left\{\exists \left[{WS}_{i},𝒩\left({WS}_{i}\right)\right]\right\}=-{WS}_{i}g-\mathrm{\backslash Updelta}g^{2}-a^{k}{WS}_{i}\\ =-{WS}_{i}\left({WS}_{i}-b^{k}\right)-\mathrm{\backslash Updelta}({WS}_{i}-b^{k}{)}^{2}-a^{k}{WS}_{i}\\ \begin{array}{c} =-{{WS}_{i}}^{2}\left(1+\mathrm{\backslash Updelta}\right)+{WS}_{i}\mathrm{\backslash Updelta}\left(1+2\mathrm{\backslash Updelta}\right)-\mathrm{\backslash Updelta}{b^{k}}^{2}-a^{k}{WS}_{i}\\ =-{{WS}_{i}}^{2}\left(1+\mathrm{\backslash Updelta}\right)+{WS}_{i}\mathrm{\backslash Updelta}\left(1+2\mathrm{\backslash Updelta}\right)-\mathrm{\backslash Updelta}{b^{k}}^{2}-a^{k}{WS}_{i}\\ \begin{array}{c} -{{WS}_{i}}^{2}+{WS}_{i}b^{k}-a^{k}{WS}_{i}\left[\text{as }\mathrm{\backslash Updelta}\text{is negligible }|\mathrm{\backslash Updelta}\to 0\right]\\ ={WS}_{i}\left(b^{k}-a^{k}\right)-{{WS}_{i}}^{2}={WS}_{i}b^{k}-{{WS}_{i}}^{2}\left[\text{as }b^{k}\geq a^{k},b^{k}\left({WS}_{i}|\mathrm{\backslash Updelta}\right)\text{is alone }\right] \end{array} \end{array} \end{array}\}$$ (12)

**Fig 4:**
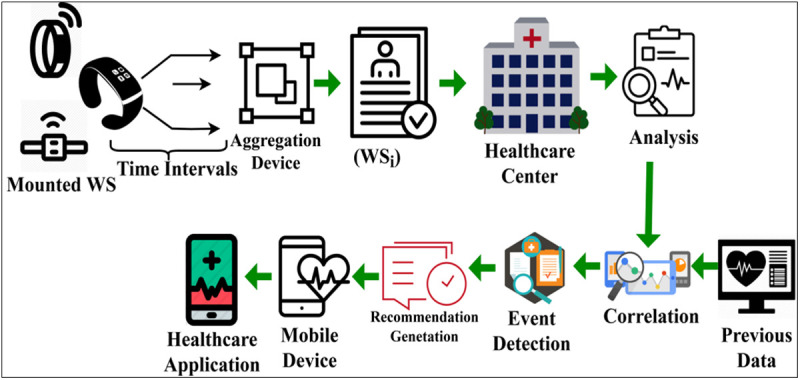
Application scenario.

Streaming precise observation instances helps improve data accumulation and reduces error and interruption. Fig 4 presents the real-time application scenario for the above method. To improve adaptability in various medical contexts, FDIM incorporates adaptive priority algorithms that modify data transmission and processing in real-time according to clinical urgency. We use a hierarchical priority scheduling system to sort the data from wearable sensors into critical, moderate, and low levels. Optimized transmission policies are learned by reinforcement learning-based priority adaptation using trends in past patient data and real-time sensor readings, further refining scheduling. While maintaining overall system efficiency, latency-aware task reallocation guarantees that high-priority data, such as aberrant vitals, gets timely processing.

In a real-time scenario, the mobile device is an aggregator from which different inputs are sensed and sent to the healthcare center. The WS, such as heartbeat sensors, headbands, wristwatches, etc., periodically senses psychological signals from the human body and shares them with the healthcare center. The healthcare center is reachable through cloud and infrastructure units. In this center, input consistency and analysis of erroneous observations are validated. The analysis is performed based on previously stored medical record data to detect any event. Such events are identified for recommendation through direct or clinical recommendations and transmitted to the mobile device.

The healthcare applications in the mobile device translate the response/ recommendation as useful information through text or multimedia messages. The aggregating device incorporates the proposed method to make it available in different instances. These instances must ensure two requisites for the clinical analysis, namely errorless data aggregation and sufficient availability. The proposed method satisfies the requirements for maximizing the correlation and precise diagnosis results. In Table 2, the normalization required for different input instances is tabulated. To adjust to changes in data distribution over time, the federated learning model in FDIM is updated periodically using an adaptive federated optimization technique. A dynamic update interval is used to avoid unnecessary communication overhead and guarantee rapid model adaption, where the aggregation frequency is changed in response to real-time data drift detection. This study combines concept drift detection methods like incremental learning and distributional similarity tests to find patterns in data collected by wearable sensors that have changed significantly. Weighted client updates and transfer learning are two examples of tailored federated learning strategies that improve responsiveness to individual patient differences.

**Table 2:** Normalization for different instances.

Input Instances	Identified Characteristics	Filtered Sequence	Normalization Required
40	12	31	−0.4
80	45	85	−0.32
120	58	96	−0.18
160	74	135	0.2
200	81	167	0.5

Table 2 presents the normalization required for different $WS_{i}$ under observed characteristics and sequences. The proposed method identifies $\overset{―}{A}$ and $\overset{―}{B}$ based on β for which different $𝒩\left(WS_{i}\right)$ is performed. Therefore, as the characteristics vary, the observing sequences vary, for which $f\left(a^{k+}\right)$ and $g\left(a^{k-}\right)$ are modified for maximizing β satisfaction. In Table 2, the β changes for different sequences are tabulated.

**Fig 5:**
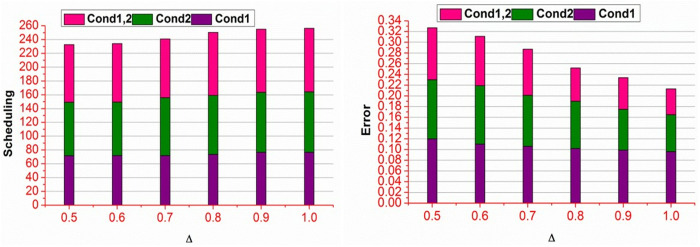
Scheduling % and error for different \Updelta.

In Table 3, the β requisites in different $a^{k}+b^{k}$ is identified based on the conditions 1 and 2. Therefore, as the $\mathrm{\backslash Updelta}>\frac{\beta }{\alpha }$ is high, then β is high due to high $𝒩\left(WS_{i}\right)$ and less ∃ observed through $g_{n}$ . This influences the $\overset{―}{A}$ and $\overset{―}{B}$ for aggregating different $WS_{i}$ without high ∃ . Thus, the requirements for β are not without maximum validations (condition 2). An analysis for scheduling % and error for different \Updelta is presented in Fig 5.

**Table 3:** .β changes for different sequences.

Sequences	Priority (Condition 1)	Priority (Condition 2)	β
30	17	12	0.53
60	26	21	0.601
90	58	36	0.685
120	74	48	0.726
150	96	53	0.841
180	103	69	0.961

**Fig 6:**
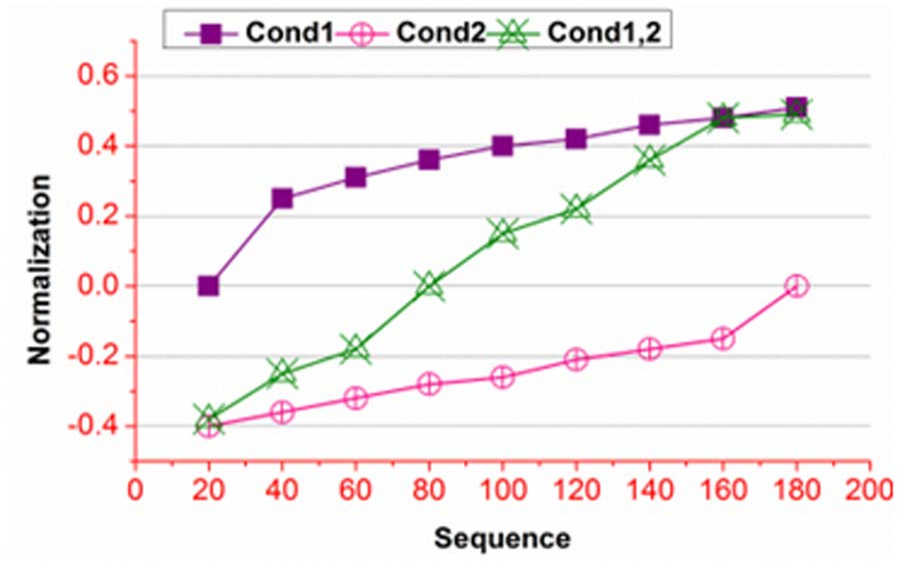
Normalization for different Sequences.

Fig 5 presents the scheduling % and error for different ∆ conditions. The conditional analysis is performed to verify priority regardless of $g\left(a^{k+}\right)$ and $g\left(a^{k-}\right)$ . These two factors vary $\overset{―}{A}$ and $\overset{―}{B}$ for reducing ∃ . In the classifier process, \Updelta based variations are defaced in reducing ∃ from equation (11) process. Therefore, the $M_{d_{\min}}=0$ is achieved for $𝒩\left(WS_{i}\right)-\mathrm{\backslash Updelta}$ in maximizing the scheduling %. As the scheduling % increases, the error decreases until $g\left(a^{k+}\right)$ and $g\left(a^{k-}\right)$ is classified using $\overset{―}{A}$ and $\overset{―}{B}$ . Fig 6 presents the normalization under different sequences.

An analysis for normalization in different sequences is presented in Fig 6. The normalization is required for ∃ experiencing instances such that β requirements are satisfied. The prolonging features depend on the $g\left(a^{k+}\right)$ and $g\left(a^{k-}\right)$ are required for $M^{bg}$ based diagnosis. Therefore, for $\mathrm{\backslash Updelta}>\frac{\beta }{\alpha },𝒩\left(WS_{i}\right)$ requires positive normalization ∀ maximization $M^{bg}$ . In the contrary case of $\mathrm{\backslash Updelta}\leq \frac{\beta }{\alpha }$ , the analysis is performed under \Updelta for reducing its impact.

## 4. Results and discussion

This section briefs the proposed method’s analysis using dataset analysis for different WS inputs. The dataset [40] provides accelerometer, gyroscope, heartbeat sensor, and EEG sensor data observed from 12 human subjects at different time intervals. The priority is set as 1 for EEG and 2 for heartbeat inputs. The data input varies from 1 MB to 16 MB in size. This input analyzes the performance using access time, complexity, data utilization, processing time, aggregation ratio, and error. In the comparative analysis, the existing TRAP [22], CDF-UA [21], and PDT-FL [26] are considered. The adaptive aggregation technique optimizes the frequency of federated learning transmission by reducing bandwidth overhead by dynamically modifying synchronization intervals depending on model divergence criteria. To ensure real-time processing, on-device execution profiling measures forward pass delays under varied compute loads to quantify local model inference latency. Utilizing low-rank decomposition and parameter quantization to decrease footprint, we assess memory usage by looking at the per-device storage allocation for model weights, gradients, and intermediate activations. To prove that FDIM can integrate EHRs in a scalable and energy-efficient manner, we do end-to-end efficiency analyses across several sensor platforms as part of our experimental validation.

### 4.1. Access time

**Fig 7:**
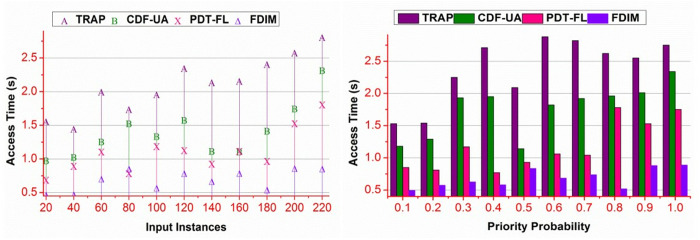
Access time analysis.

Fig 7 presents the comparative analysis of access time for input instances and priority probabilities. The proposed method achieves less access time for different \Updelta and priority estimations. In this method, the $WS_{i}$ is split based on $\overset{―}{A}$ and $\overset{―}{B}$ that validates $a^{k}+b^{k}=WS_{i}$ using $𝒩\left(WS_{i}\right)$ . Therefore, the ∃ is reduced under different validation classes as performed using the classifier learning; contrarily, the $b^{k}=0$ instances are segregated for less processing in maximizing the aggregation ratio. This enhances the available data ∃ and is ready for utilization. The available data is readily available for healthcare application support. The indistinct $g\left(a^{k+}\right)$ and $g(a^{k-})$ classified through $g_{1}$ to $g_{n}$ in the classification, instances are reduced ∃ for the aggregated data. Therefore, unavailability or erroneous processing is not required for this method. This enhances the data utilization in less access time for different \Updelta .

### 4.2. Complexity

**Fig 8:**
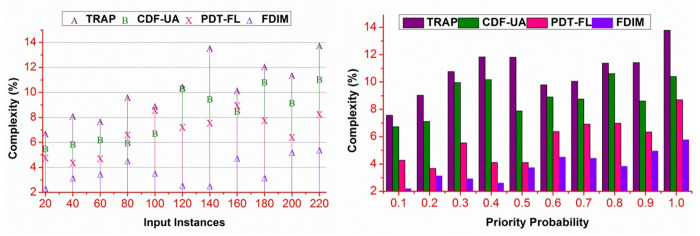
Complexity analysis.

The proposed method achieves less complexity than the other methods presented in Fig 8. The proposed method reduces ∃ based on $a^{k}\left(g|WS_{i}\right)$ and $\left(WS_{i}|d\right)$ . In both validations, the \Updelta is identified using $\left(n*\alpha_{i}\right)$ execution. This is required for distinguishing \Updelta under $>\frac{\beta }{\alpha }\text{ or}\leq \frac{\beta }{\alpha }$ under different estimations. In multiple classification instances, the above conditions are validated for $WS_{i}$ and $𝒩\left(WS_{i}\right)$ . The proposed method extracts the $\overset{―}{A}$ and $\overset{―}{B}$ in different $g_{1}$ to $g_{n}$ based on the previous $g\left(a^{k+}\right)$ and $g\left(a^{k-}\right)$ . In multiple classification instances, $\left(WS_{i}|\mathrm{\backslash Updelta}g\right)$ and (β−\Updeltag) are isolated for providing less computation-based validations. Therefore, if $b^{k}\geq a^{k}$ is achieved, then $\left(WS_{i}|\mathrm{\backslash Updelta}\right)$ is alone validated, preventing additional computation and reducing complexity under different input instances and priority predictions.

### 4.3. Data utilization

**Fig 9:**
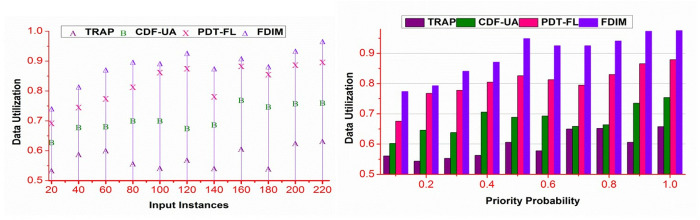
Data utilization analysis.

The proposed method improves data utilization by reducing ∃ different classification instances. The $WS_{i}$ is disintegrated using $\overset{―}{A}$ and $\overset{―}{B}$ for different $g\left(a^{k+}\right)$ and $g\left(a^{k-}\right)$ in reducing the erroneous date. As the $b^{k}=+1/-1$ varies, as do the assignment and aggregation for data availability. For different data input instances, the classification process, $M^{bg}$ based on availability and ∃ reduces its impact on the accumulated data. This intern requires an organized allocation of the sequences as defined in equation (6). The subsequent \Updelta from the classification process organizes $𝒩\left(WS_{i}\right)$ based on β such that ∃ is reduced in $M_{d}\in M_{D}$ . This enhances the utilization based on availability; hence, the conditional analysis reduces access complexity. The process achieves high data utilization for the identified α and β conditions (Fig 9).

### 4.4. Processing time

**Fig 10:**
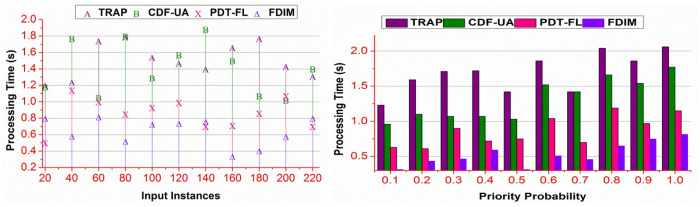
Processing time analysis.

The proposed method reduces processing time for different data inputs and priority probability (Fig 10). The proposed method classifies prioritized and non-prioritized \Updelta for reducing the processing time. The $a^{k}$ and $b^{k}$ organization reduces the ∃ occurrence regardless of multiple instances provided $M_{d}\in M_{D}$ . In the alternating validation, verification of $b^{k}=\frac{\beta }{\alpha }$ is performed. This validation reduces the $b^{k}\left(WS_{i}|\alpha \right)=\gamma \left(\alpha^{k}-\mathrm{\backslash Updelta}g\right)$ validation, preventing the additional data processing steps. In the complemented processing instances, if $\mathrm{\backslash Updelta}>\frac{\beta }{\alpha }$ or $\mathrm{\backslash Updelta}\leq \frac{\beta }{\alpha }$ true, then either of the conditions is alone validated. Therefore, the processing is performed for $g_{1}$ to $g_{n}\in \left(\frac{\exists }{M_{D}}\right)$ or $g_{1}\in \left(\frac{\mathrm{\backslash Updelta}*\exists }{M_{D}}\right)$ . Thus, the processing is allocated depending on the ∃ classification used to distinguish prioritized requirements. Therefore, the classification processing for both instances is distinguished under controlled time.

### 4.5. Aggregation ratio

**Fig 11:**
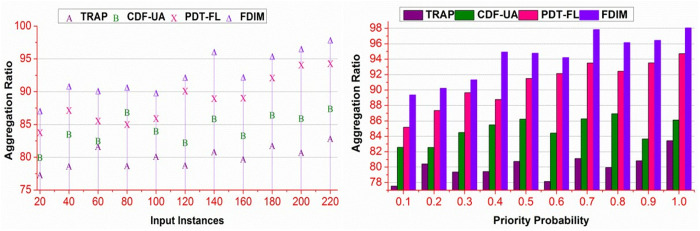
Aggregation ratio analysis.

The proposed method achieves a high aggregation ratio compared to the other methods. The input data instances are organized based on proceeding $𝒩\left(WS_{i}\right)+\frac{\beta }{\alpha }$ such that stagnancy is less. In the different processing instances, the \Updelta based modifications are performed without reducing the incoming data. In contrast to the aggregated sequences of $a^{k}+b^{k}=WS_{i}$ , the $M^{bg}$ validation increases the validation based on ∃ it, and thus, the unsalted incoming is retained. Therefore, the classifier is trained to validate $a^{k}\left(WS_{i}|\alpha \right)$ and $a^{k}\left(g|WS_{i}\right)$ in different prediction instances. The consecutive instances are validated for indistinct $\frac{\beta }{\alpha }$ and $\frac{\exists }{M_{D}}\forall M_{d}\in M_{D}$ for preventing input stagnancy. The contrast formulation deviated based on $M_{d_{\min}}=0$ and $M_{d}=M_{D}$ such that $\frac{\rho_{r}}{\rho_{\beta }}=1$ is achieved. In the proposed method, the available assessments are performed without preventing $b^{k}\geq a^{k}$ and leaving and $\left(WS_{i}|\mathrm{\backslash Updelta}\right)$ validation. Therefore, the aggregation for filling up the $\frac{\beta }{\alpha }$ difference is utilized to increase the aggregation ratio. The proposed method regains the aggregation for different data inputs and priorities (Fig 11).

### 4.6. Error

**Fig 12:**
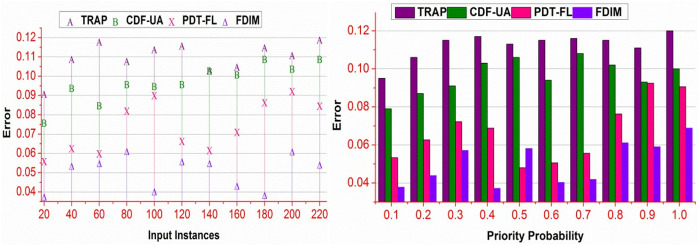
Error analysis.

The proposed method is confined ∃ to different data instances and prediction probabilities. The proposed method performs v estimation for the detection of abnormal events. In the $𝒩\left(WS_{i}\right)$ , the available classifications are validated without reducing the occurrence. The further deviations in $M_{d}\in M_{D}$ are distinguished as $M_{D_{\max}}$ and $M_{D_{\min}}$ for preventing summed errors. The β distinguishing at regular intervals of the above process reduces $\left(WS_{i}-\overset{―}{WS_{i}}\right)$ computations. In the contrary process, $\overset{―}{A}$ and $\overset{―}{B}$ validations are performed $\forall g\left(a^{k+}\right)$ and $g\left(a^{k-}\right)$ such that gA→B is validated in $M_{d}$ alone. Therefore, the consecutive process is identified from $b^{k}=-1$ to (α=β)=0 is normalized. This reduces the ∃ in the first classification instance, preventing priority interruption. The contrast validations are performed for $\mathrm{\backslash Updelta}\leq \frac{\beta }{\alpha }$ and hence the $\left(WS_{i}|\mathrm{\backslash Updelta}\right)$ is alone assessed for reducing further errors. In the second classification using random forests, $M_{d}=M_{D}$ is identified to prevent further errors. This is pervasive for different $WS_{i}$ and \Updelta as presented in Fig 12. Table 4 and Table 5 summarize the above comparative analysis for input instances and priority probability.

**Table 4:** Comparative analysis for input instances.

Metrics	TRAP	CDF-UA	PDT-FL	Proposed	Findings
Access Time (s)	2.81	2.32	1.81	0.859	10.49% Less
Complexity (%)	13.77	11.07	8.27	5.428	5.61% Less
Data Utilization	0.633	0.761	0.897	0.967	10.17% High
Processing Time (s)	1.31	1.4	0.7	0.801	9.81% Less
Aggregation Ratio	82.91	87.46	94.35	97.959	9.72% High
Error	0.119	0.109	0.0848	0.0543	9.9% Less

**Table 5:** Comparative analysis for priority probability.

Metrics	TRAP	CDF-UA	PDT-FL	Proposed	Findings
Access Time (s)	2.75	2.34	1.75	0.887	10.18% Less
Complexity (%)	13.79	10.4	8.7	5.769	5.19% Less
Data Utilization	0.658	0.754	0.879	0.975	10.57% High
Processing Time (s)	2.06	1.77	1.15	0.815	8.48% Less
Aggregation Ratio	83.43	86.11	94.72	98.056	9.97% High
Error	0.12	0.1	0.0906	0.0688	10.42% Less

### 4.7. Ablation study

Ablation studies are conducted by systematically deactivating specific FDIM modules to quantify their independent contributions to performance metrics, including communication efficiency, inference latency, and energy consumption. The study isolates Mobile Bridge (MB), Time Interval Interrupts (TII), Multi-Bridging (MBR), and Processing Device Scheduling (PDS) as key components.

1. **Baseline Comparison:** FDIM is fully configurable to establish benchmark performance across latency, power efficiency, and data throughput.
2. **Module Removal Strategy:** Each component is selectively disabled while maintaining all other configurations constant.
  - **Without MB:** Direct transmission from wearable sensors to processing devices is tested, analyzing the impact on data congestion and computational overhead.
  - **Without TII:** Continuous data flow is enforced without interruption-based scheduling, measuring the effect on transmission stability and power consumption.
  - **Without MBR:** Single-server bridging is enforced, and network load distribution and failover efficiency are evaluated.
  - **Without PDS:** FIFO-based data handling replaces priority-based and linear scheduling, assessing its influence on real-time processing.
3. Performance Metrics: Each variation is tested across multiple trials, measuring power draw per cycle (mW), average inference time (ms), packet loss rate (%), and federated model convergence stability.

The results quantify the significance of each module, demonstrating that TII and PDS contribute most to energy efficiency and latency reduction, while MBR enhances fault tolerance and throughput. These findings validate FDIM’s modular effectiveness in wearable sensor data aggregation for electronic health records.

The efficiency of FDIM is evaluated in real-world deployments, where the operation of continuous wearable sensors is affected by limitations on battery life and is integrated power consumption comparisons. Data transmission, computational load, and dynamic power use efficiency are measured using energy profiling and compared to preexisting frameworks like TRAP, CDF-UA, and PDT-FL. The analysis of power consumption in real-time, optimization of duty cycles, and use of adaptive resource allocation are all part of the experimental assessment. The findings demonstrate that FDIM is feasible for clinical-grade EHR augmentation and quantifies its energy efficiency, ensuring that it will continue to work in wearable devices connected to hospitals.

To improve EHRs, the Fair Dividend Interrupt Method (FDIM) integrates data from several wearable sensors via a fair data fusion process that equalizes the representation of physiological characteristics and reduces bias. However, validation methods often rely on artificial datasets, which severely limit their usefulness in healthcare environments. Real-world pilot studies evaluate the efficacy of FDIM in collecting data in real-time, detecting anomalies, and making predictions by integrating it with live patient monitoring frameworks and deploying it within the interfaces of hospital information systems. Measures include data quality, latency, impact on decision support systems, and integration with current clinical processes to assess how well it optimizes patient outcomes and enhances EHR dependability.

## 5. Conclusion

Wearable sensor data organization and aggregation is challenging due to asynchronous device operations and priorities. This paper discussed a fair dividend interrupt method for improving wearable sensor observations’ aggregation and data utilization probability. The distinguished input instances are sequentially validated to extract the computing characteristics for multi-bridging input diagnosis. In this process, prioritized conditional analysis augments the scheduling process without reducing the data utilization. The federated learning process decides efficiency verification based on error inputs and aggregation outputs. The scheduled data is verified for its priority in distinct intervals, augmenting high aggregation. The proposed method is reliable in assisting healthcare applications through precise data aggregation and medical risk assessment. From the experimental results, the proposed method is found to achieve 10.18% less access time, 5.19% less complexity, 10.57% high data utilization, 8.48% less processing time, 9.97% high aggregation ratio, and 10.42% less error for different priority probabilities. Future work include applying this method to various WS sites and organizations outside the healthcare industry. In addition, the federated learning process strives to become even more skilled and accurate in handling various inputs. This requires ongoing improvement and a concentration on enhancing decision-making methods.
